# Monitoring the Prevalence of *Leucocytozoon sabrazesi* in Southern China and Testing Tricyclic Compounds against Gametocytes

**DOI:** 10.1371/journal.pone.0161869

**Published:** 2016-08-29

**Authors:** Wenting Zhao, Qin Pang, Ruixue Xu, Jianwen Liu, Shengfa Liu, Jian Li, Xin-zhuan Su

**Affiliations:** 1 State Key Laboratory of Cellular Stress Biology, Innovation Center for Cell Signaling Network, School of Life Sciences, Xiamen University, Xiamen, Fujian 361005, P. R. China; 2 Laboratory of Malaria and Vector Research, National Institute of Allergy and Infectious Diseases, National Institutes of Health (NIH), Bethesda, Maryland 20892, United States of America; Institute of Tropical Medicine, JAPAN

## Abstract

*Leucocytozoon* parasites infect many species of avian hosts, including domestic chicken, and can inflict heavy economic loss on the poultry industry. Two major species of *Leucocytozoon* parasites have been reported in China, *L*. *sabrazesi* and *L*. *caulleryi*, although *L*. *sabrazesi* appears to be more widespread than *L*. *caulleryi* in southern China. The traditional method for detecting *Leucocytozoon* infection is microscopic examination of blood smears for the presence of mature gametocytes in circulation, which may miss infections with low parasitemia (gametocytemia) or immature gametocytes. Here we developed a PCR-based method to monitor *L*. *sabrazesi* infections at seven sites in four provinces of China after testing two PCR primer pairs based on parasite mitochondrial cytochrome b (*cytb*) and cytochrome c oxidase III (*coxIII*) genes. We compared the results of PCR detection with those of microscopic observation. As expected, the PCR assays were more sensitive than microscope examination in detecting *L*. *sabrazesi* infection and were able to detect parasite DNA after gametocytes disappeared in the blood stream. Using these methods, we investigated monthly dynamics of *L*. *sabrazesi* in chickens from a free-range farm in Xiamen, Fujian province of China, over one year. Our results showed that chickens were infected with *L*. *sabrazesi* year-round in southern China. Finally, we tested several compounds for potential treatment of *Leucocytozoon* infections, including primaquine, ketotifen, clomipramine hydrochloride, desipramine hydrochloride, sulfaquinoxaline, and pyrimethamine. Only primaquine had activity against *L*. *sabrazesi* gametocytes. Our results provide important information for controlling parasite transmission in southern China and disease management.

## Introduction

*Leucocytozoon* parasites infect numerous species of avian hosts, including domestic chickens [1–7]. The parasites belong to a genus of parasitic protozoa in the phylum Apicomplexa that also contains parasites causing human malaria and Toxoplasmosis. The *Leucocytozoon* parasites have a complex life cycle involving two hosts, having merogony in fixed tissues (such as liver) and sexual stages (gametocytes) in blood cells of vertebrate hosts, and sporogony in simuliid flies (Simuliidae) or culicoides midges [1]. Infection with *Leucocytozoon* parasites could result in increased chicken mortality and decreased egg production, with symptoms of listlessness, green feces, anorexia, anemia, and even death [1, 8–10]. Detrimental effects on host reproductive success, host fitness, and economic losses by infections of *Haemoproteus* and/or *Leucocytoazoon* parasites have been reported [11–13]. Infected domestic chickens could have mortality rates as high as 56%, leading to significant economic loss [14]. However, it was also reported that *Leucocytozoon simondi* did not affect the growth rates of infected ducklings [15].

Treatment of *Leucocytozoon* infection is still difficult, although various antimalarial and anticoccidial compounds have been reported to have effects on bird blood protozoans such as *Leucocytozoon* parasites, including clopidol [16], primaquine [11, 15], and halofuginonepolystyrene sulfonate [17]. Additionally, sulfamonomethoxine and ormetoprim combinations were reported to prevent or reduce *Leucocytozoon* infection [18]. However, paludrine, atebrin, and sulphamerazine had no activities against *L*. *simondi* in ducklings [19]. Development of additional safe, effective, and affordable compounds for treating *Leucocytozoon* gametocytes to prevent transmission is necessary to control the disease.

Previous surveys of *Leucocytozoon* infections in China were mostly based on species identification of the circulating gametocytes in blood smears [14, 20–23], although a polymerase chain reaction (PCR) method based on ribosomal DNA ITS2 sequence to detect *L*. *caulleryi* was described previously [24]. These studies have provided important information for the understanding of the disease prevalence and for disease control; however, the fact that most of *L*. *sabrazesi* infections have low parasitemia (Because only gametocytes are detected in the blood, parasitemia is equal to gametocytemia) can lead to false negatives in diagnosis based on microscopic examination. Detection of parasite DNA or RNA using PCR may inform potential infections even when blood smears are negative, and can improve the sensitivity of detecting parasites in a host. Indeed, there have been reports of detecting infections of *Leucocytozoon* and other blood protozoan parasites in avian and insect hosts using PCR methods employing primers derived from parasite genes such as the cytochrome b (*cytb*) gene [25–31]. For example, using primers from a segment of the *cytb* gene, Ortego and Cordero (2009) amplified DNA from blood samples of 206 nestling eagle owls (Bubo bubo) in the Toledo province of Central Spain and revealed a unique lineage of *Leucocytozoon ziemanni* in the eagle owls [26]. The *cytb* gene of *Leucocytozoon lovati* was also employed to study DNA from 490 insect samples of six black fly species [25]. Similarly, by amplifying a 478 bp fragment from the *cytb* gene, Ferraguti et al. (2013) identified six *Haemoproteus* and two *Plasmodium* lineages of blood parasites in 13 biting midges (out of 97 tested) and in 123 bird blood samples [28]. These studies suggest that PCR amplification of parasite mitochondrial DNA is a reliable approach for detecting blood protozoan parasites, including *Leucocytozoon spp*.

In our previous study, we showed that domestic chickens in southern China were often infected with multiple strains of *L*. *sabrazesi* and that gametocytes could “relapse” or be persistent at low-level for 4–5 months without active transmission [23], suggesting potential year-round transmission if insect vectors are available. More recently, we showed that the gametocytes of *L*. *sabrazesi* infected chicken thrombocytes [32], but not other blood cells. Erythroblasts, red blood cells (RBCs), and mononuclear leukocytes have historically been reported to be the host cells of *Leucocytozoon* gametocytes [33–36]. To better understand the transmission dynamics in field settings in southern China, we investigated year-round *L*. *sabrazesi* infection found at a free-range farm by collecting chicken blood samples monthly from October 2014 to September 2015. We also designed and evaluated two pairs of PCR primers based on the parasite mitochondrial *cytb* and cytochrome c oxidase III (*coxIII*) genes and compared the results from the PCR methods with those of microscopic examination. We found that negative blood smear samples were often positive by PCR methods. Our results also showed the presence of parasites and/or parasite DNA in chicken blood samples collected year-round, with the highest prevalence in June. Finally, we tested several compounds, including some that were shown to block transmission of *Plasmodium* parasites [37], for potential treatment of *Leucocytozoon* infections. These results provide important information for better disease control and management.

## Materials and Methods

### Ethics Statement

Chickens were purchased from the farm owners or with permission from the owners before blood drawing. The experiments were conducted under a protocol (protocol #XMULAC20130065) approved by the Animal Care and Ethics Committee at Xiamen University.

### Sampling sites and blood collection

Chicken blood samples were collected from three sites in Fujian province (Haicang, geographic coordinates 24.4845° N, 118.0328° E; Zhangzhou, 24.5130° N, 117.6471°E; Fuzhou; 26.0745° N, 119.2965° E), two sites in Jiangxi province (Ganzhou, 25.8318° N, 114.9350° E; Ruijin, 25.8856° N, 116.0271° E), one site in Sichuan province (Dazhou, 31.2096° N, 107.4680° E), and one site in Shandong province (Binzhou, 37.3820° N, 117.9707° E) of China as previously described [23]. The chickens (*Gallus gallus domesticus*) were raised either in free-range farms or in the backyards of individual households. Two to three hundred microliters of blood was collected from the wing veins and was immediately mixed with anticoagulant (8 g/L citric acid and 22 g/L trisodium citrate, pH 7.2) in a 1.5 ml centrifuge tube. Thin blood smears were made, air-dried, fixed with 100% methanol, and stained with Giemsa stain. Parasitemia (or gametocytemia in this case) were estimated as the proportion of infected cells after counting 10,000 red blood cells (RBCs).

### DNA extraction

Blood cells were lysed using saponin solution before DNA extraction. Briefly, 10–20 μl packed blood cells were suspended in 20 volumes of 0.1% saponin in PBS (pH 7.4) and kept on ice for 5 min. The parasites and nuclei of host cells were pelleted by centrifugation at 5000 rpm for 3 min and then washed with PBS (3X). The pellets were re-suspended in 400 μl lysis buffer (50 mM TrisCl, 100 mM EDTA, 2% SDS; pH 8.0, 100 μg/ml proteinase K), and incubated overnight at 50°C. DNA was extracted using the phenol-chloroform method and dissolved in ddH_2_O.

### DNA amplification and PCR assays

**Fig 1:**
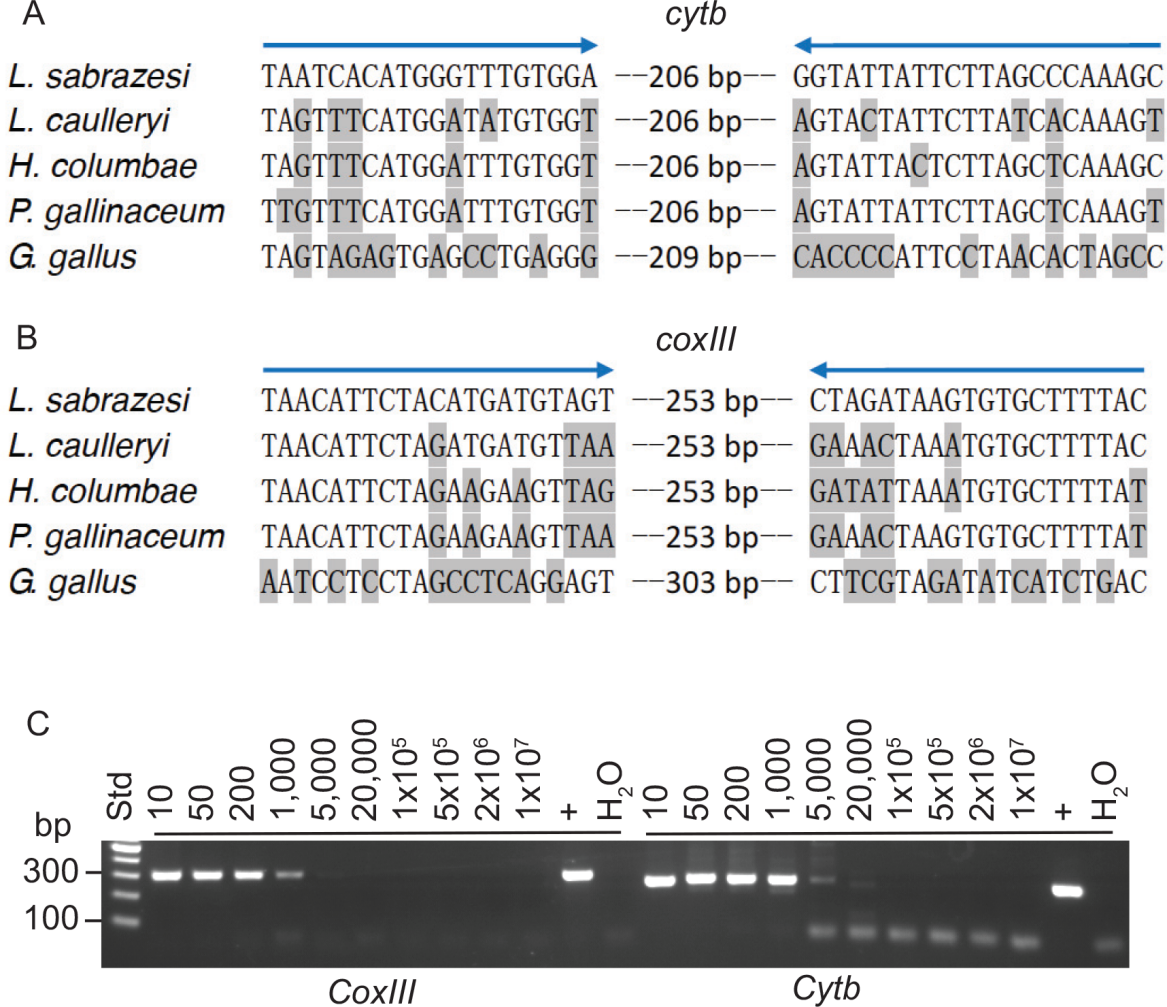
Primer design and detection of diluted DNA samples from an infected chicken. **A** and **B,** Aligned primer sequences based on genes encoding mitochondrial cytochrome b (*cytb*, **A**) and cytochrome oxidase subunit III (*coxIII*, **B**) from *L*. *sabrazesi* (NCBI accession No. AB299369.1), *Leucocytozoon caulleryi* (Accession No. AB302215.1), *Haemoproteus columbae* (NCBI accession No. FJ168562.1), *Plasmodium gallinaceum* (Accession No. AB250690.1), and chicken (*Gallus gallus domesticus*, Accession No. KM096864.1). **C**, Amplifications of diluted DNA from an infected chicken with known gametocytemia. DNA sample from infected chicken (#2HC with parasitemia of 0.02%) blood obtained from Haicang, Fujian province, was diluted in water at ratios of 1:10; 1:50; 1:200; 1:1,000; 1:5,000; 1:20,000; 1:100,000; 1:500,000; 1:2×10^6^; and 1:1×10^7^ and was amplified using Ls-coxIIIF2/R2 and Ls-cytbF1/R1 primers, respectively. PCR products (4 μl each) were separated on a 2% agarose gel. A DNA band could be easily detected at 1:1,000 dilution using Ls-coxIIIF2/R2, and a band at 1:5,000 could be seen using the Ls-cytbF1/R1 primers. “+” indicates un-diluted DNA control. The figure is representative of two replicates with the same results.

Two pairs of primers were designed based on the mitochondrial sequence encoding *cytb* and *coxIII* genes: Ls-cytbF1(5’taa tca cat ggg ttt gtg ga3’) and Ls-cytbR1(5’gct ttg ggc taa gaa taa tac c3’), and Ls-coxIIIF2(5’taa cat tct aca tga tgt agt3’) and Ls-coxIIIR2(5’gta aaa gca cac tta tct ag3’) from *cytb* and *coxIII* gene, respectively. Oligonucleotide primers were designed to specifically amplify *cytb* and *coxIII* genes based on the mitochondrial DNA sequence of *L*. *sabrazesi* (NCBI accession No. AB299369.1). The *L*. *sabrazesi* mitochondrial *cytb* and *coxIII* gene sequences were aligned with those from representative avian blood parasites *Leucotytozoon caulleryi* (Accession No. AB302215.1), *Haemoproteus columbae* (NCBI accession No. FJ168562.1), *Plasmodium gallinaceum* (Accession No. AB250690.1), and the sequences of chicken (*Gallus gallus domesticus*) mitochondrial DNA (Accession No. KM096864.1). For each primer, there was a minimum of 3 base pair mismatches, including the 3’end nucleotide, to ensure amplification specificities (**Fig 1A and 1B**). The primers were synthesized by a commercial company (Sangon Biotech Co., Ltd., Shanghai). All PCR amplifications were performed in a 20 μl volume containing 4 μl of genomic DNA (~50 pg), 80 mM dNTPs, 0.8 mM MgCl_2_, 0.1 mM of each primer, and 1 unit of Taq DNA polymerase. The primers Ls-cytbF1 and Ls-cytbR1 amplified a fragment of 248 basepair (bp) in the *cytb* gene. The cycling conditions were: initial denaturation at 94°C for 2 min; cycling 94°C for 20s, 53°C for 20s, 68°C for 30s for 40 cycles; and a final step at 68°C for 2 min. The Ls-coxIIIF2 and Ls-coxIIIR2 primers amplified a DNA fragment of 294 bp; the cycling conditions were: initial denaturation at 94°C for 2 min; cycling 94°C for 20s, 50°C for 20s, and 68°C for 30s for 40 cycles; and a final step at 68°C for 2 min. The PCR products were separated on 2% agarose gels, visualized on a UV light box, and photographed using a Tanon-2500(R) Gel Imaging System (Tanon).

### Drug treatment procedures

Female chickens (*Gallus gallus domesticus*, six to 12 months old) infected with *L*. *sabrazesi* were confirmed by microscopic observation and PCR assays and then randomly divided into groups of two each for drug tests. The chickens were housed in wired cages within a room and were fed with commercial chicken food with balanced nutrition from Liaocheng Shunda Feedstuff Co., Ltd, China. The room was cleaned daily, and their droppings, appetite, and activity were monitored at the same time. Pyrimethamine were dissolved in DMSO; primaquine, ketotifen, sulfaquinoxaline, clomipramine hydrochloride, and desipramine hydrochloride were dissolved in water and stored at -20°C until use. The compounds were obtained from Sigma-Aldrich (St. Louis, MO). Chickens were given once-daily oral treatment for 14–20 consecutive days and were kept in a room with screened windows. Chickens receiving no drug treatment served as the control group. Blood samples were collected from chickens at 3- to 6-day intervals for one to two months during and post drug treatment for microscopic examination and for DNA extraction.

## Results

### Evaluation of PCR primer sets for the detection of parasite DNA

To develop a reliable and sensitive method to detect parasite DNA in the blood, we synthesized two pairs of primers based on *L*. *sabrazesi* mitochondrial *cytb* and *coxIII* genes, respectively (Ls-cytb F1/R1 and Ls-coxIII F2/R2). The primer sequences were selected based on the aligned sequences of mitochondrial *cytb* and *coxIII* genes of *L*. *sabrazesi* and those of common avian blood parasites such as *Haemoproteus* and/or *Leucocytozoon spp* (**Fig 1A** and **1B**). We first compared the sensitivity of the primer pairs in detecting diluted DNA samples from an infected chicken that had a known parasitemia (0.02%) and showed that both primer sets could detect parasite DNA diluted up to1,000–5,000 fold, with the *cytb* primers appearing to be slightly more sensitive (a faint band could be seen at 1:20,000 dilution) than the *coxIII* primers in detecting parasite DNA (**Fig 1C**). Amplification of water without DNA or uninfected chicken blood cells (see below) did not produce any DNA bands. The results suggest that the PCR methods could detect infection with a parasitemia as low as 2 gametocytes per 1×10^5^ RBCs for *coxIII* primers and 2 gametocytes per 5×10^5^ RBCs for the *cytb* primers.

### Comparison of microscopy and PCR detection

**Fig 2:**
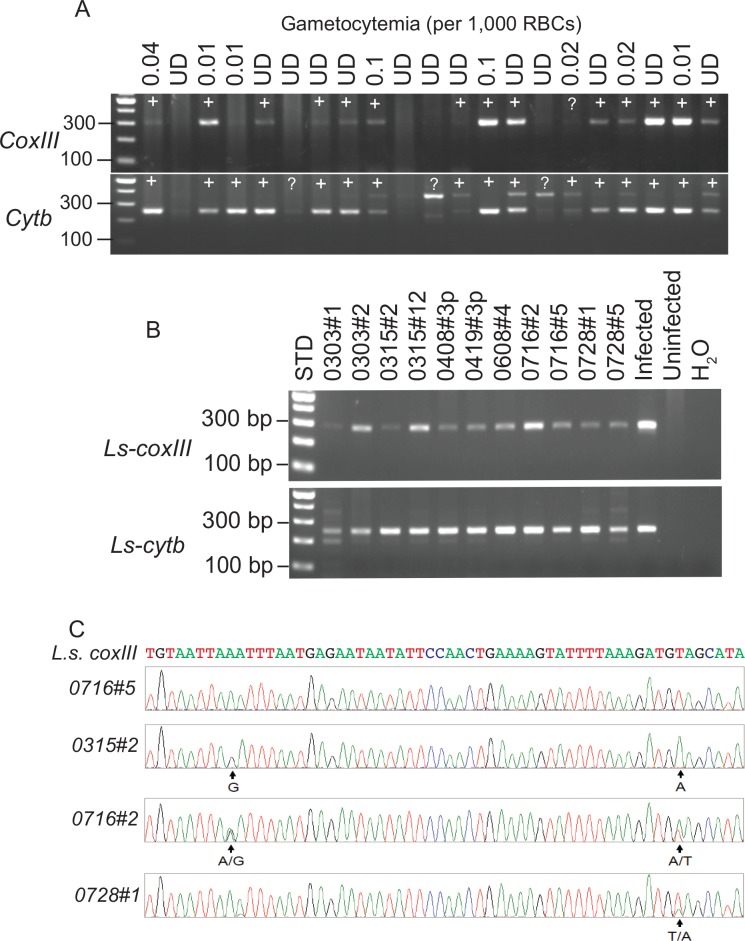
PCR detection of parasite DNAs extracted from chicken blood samples with or without positive identification of gametocytes. **A**, PCR products amplified using the Ls-coxIIIF2/R2 and Ls-cytbF1/R1 primers, respectively. The numbers on top of the gels are gametocytemia per 1,000 red blood cells (RBCs) after counting 10,000 RBCs. DNAs were extracted from 20 μl infected blood, amplified using the Ls-coxIIIF2/R2 and Ls-cytbF1/R1 primers, respectively, and separated on a 2% agarose gel (4 μl PCR products loaded). DNA ladders of one hundred bp (100–500 bp) are on the left side of the gels. “+” indicates PCR positive; “?” positive with uncertainty; UD, undetectable gametocyte; The figure is representative of two replications of the same results. **B**, PCR products from microscopic negative samples amplified using the Ls-coxIIIF2/R2 and Ls-cytbF1/R1primers, respectively. **C**, Electropherograms from four microscopic negative samples by Ls-coxIIIF2/R2 primers showing double peaks in two samples and two nucleotide substitutions in one sample (0315#2).

We next used the primers to detect DNA samples from chickens that were microscopic positive or negative. Consistent with the results from diluted DNA samples, the Ls-cytbF1/R1 primers were more sensitive in detecting DNA in infected chickens, including some samples that were negative in blood smears (**Fig 2A**), whereas the *coxIII* primers appeared to be more specific, consistently producing a single band of expected size.

To further confirm the specificity of the PCR methods, we amplified 11 samples that were microscopic negative using the Ls-coxIIIF2/R2 and Ls-cytbF1/R1 primers. Bands of expected sizes were obtained from both primer sets, although additional bands were detected in some samples by the Ls-cytbF1/R1 primers (**Fig 2B**). We sequenced the PCR products from the Ls-coxIIIF2/R2 primers directly without cloning into a plasmid vector and obtained 11 sequences, including five sequences that appeared to have infections of more than one strain (double electropherogram peaks at one position) (**Fig 2C** and **S1 Fig**). The high frequency of mixed infections is consistent with our previous observations [23]. We next cloned the PCR products into Pmd18-T vector for sequencing and obtained 24 individual sequences; again, all the sequences matched the *coxIII* sequence of *L*. *sabrazesi* (**S2 Fig**). Clustering the amplified sequences with those of representative blood parasites showed that they closely matched the sequence of *L*. *sabrazesi* (**S3 Fig**). These results at least confirmed the specificity of the Ls-coxIIIF2/R2 primers for *L*. *sabrazesi* detection [23].

We next amplified additional DNA samples (a total of 102) from chickens previously collected from seven locations in four provinces in China [23] using the Ls-coxIIIF2/R2 primers (these primers were used to be conservative for PCR positives). As expected, more samples were positive by PCR amplification than microscopic observation (**Table 1**). Among the 102 samples, 45 were positive by microscopy, and all were positive by the Ls-coxIIIF2/R2 PCR method. Among the 57 negative blood smear samples after counting 10,000 RBCs, 13 were positive by PCR (**Table 1**), providing a positive rate of 56.9%. The results also supported that the PCR method was more sensitive than blood smear in detecting *Leucocytozoon* infection.

**Table 1:** Comparison of microscopic examination of blood smear and the Ls-coxIIIF2/R2 PCR method in detecting *Leucocytozoon sabrazesi* infection.

Date	Sample No.	Gametocytemia (%)	PCR detection	Date	Sample No.	Gametocytemia (%)	PCR detection
11-Dec-12	ZZ-1#1	0.004	+	5-Jul-13	HC-3#4	0.001	+
11-Dec-12	ZZ-1#2	0	-	5-Jul-13	HC-3#5	0.0067	+
11-Dec-12	ZZ-1#3	0.011	+	5-Jul-13	HC-3#6	0.0047	+
11-Dec-12	ZZ-1#4	0	-	5-Jul-13	HC-3#7	0.0168	+
11-Dec-12	ZZ-1#5	0.0003	+	29-Mar-13	FZ-1#1	0	-
11-Dec-12	ZZ-1#6	0	-	29-Mar-13	FZ-1#2	0	+
11-Dec-12	ZZ-1#7	<0.0001	+	29-Mar-13	FZ-1#3	0	+
11-Dec-12	ZZ-1#8	0	-	29-Mar-13	FZ-1#4	0	+
11-Dec-12	ZZ-1#9	0.0007	+	29-Mar-13	FZ-1#5	0	-
11-Dec-12	ZZ-1#10	<0.0001	+	29-Mar-13	FZ-1#6	0	+
11-Dec-12	ZZ-1#11	<0.0001	+	29-Mar-13	FZ-1#7	0	+
11-Dec-12	ZZ-1#12	<0.0001	+	29-Mar-13	FZ-1#8	0	+
11-Dec-12	ZZ-1#13	0	-	29-Mar-13	FZ-1#9	0	+
11-Dec-12	ZZ-1#14	0.0003	+	29-Mar-13	FZ-1#10	0	-
11-Dec-12	ZZ-1#15	0.005	+	29-Mar-13	FZ-1#11	0	-
11-Dec-12	ZZ-1#16	0	-	9-Feb-13	GZ#1	0.0022	+
11-Dec-12	ZZ-1#17	0	-	9-Feb-13	GZ#2	0	-
11-Dec-12	ZZ-1#18	0.009	+	9-Feb-13	GZ#3	0.0046	+
11-Dec-12	ZZ-1#19	0.007	+	9-Feb-13	GZ#4	0	+
11-Dec-12	ZZ-1#20	0.009	+	9-Feb-13	GZ#5	0.0033	+
11-Dec-12	ZZ-1#21	0.003	+	9-Feb-13	GZ#6	0.0045	+
11-Dec-12	ZZ-1#22	<0.0001	+	9-Feb-13	GZ#7	0.0038	+
11-Dec-12	ZZ-1#23	0	-	9-Feb-13	GZ#8	0	+
11-Dec-12	ZZ-1#24	0.022	+	10-Mar-13	RJ#1	0	-
11-Dec-12	ZZ-1#25	<0.0001	+	10-Mar-13	RJ#2	0	-
25-Aug-13	ZZ-2#1	0	+	10-Mar-13	RJ#3	0	-
25-Aug-13	ZZ-2#2	0	+	10-Mar-13	RJ#4	0	-
25-Aug-13	ZZ-2#6	0.018	+	10-Mar-13	RJ#5	0	-
25-Aug-13	ZZ-2#7	0.03	+	10-Mar-13	RJ#6	0	-
25-Aug-13	ZZ-2#8	0.0043	+	10-Mar-13	RJ#7	0	-
25-Aug-13	ZZ-2#9	0.0057	+	10-Mar-13	RJ#8	0	-
25-Aug-13	ZZ-2#11	0.0057	+	10-Mar-13	RJ#9	0	-
25-Aug-13	ZZ-2#13	0	+	10-Mar-13	RJ#10	0	-
25-Aug-13	ZZ-2#14	0.0011	+	8-Feb-13	DZ#1	0	-
25-Aug-13	ZZ-2#19	0.0035	+	8-Feb-13	DZ#2	0	-
23-Mar-13	HC-1#1	0.0184	+	8-Feb-13	DZ#3	0	-
23-Mar-13	HC-1#2	0.0492	+	8-Feb-13	DZ#4	0	-
23-Mar-13	HC-1#3	0.013	+	8-Feb-13	DZ#5	0	-
23-Mar-13	HC-1#4	0.0408	+	8-Feb-13	DZ#6	0	-
23-Mar-13	HC-1#5	0	+	8-Feb-13	DZ#7	0	-
23-Mar-13	HC-1#6	0	-	8-Feb-13	DZ#8	0	-
23-Mar-13	HC-1#7	0.0252	+	8-Feb-13	DZ#9	0	-
23-Mar-13	HC-1#8	0.0108	+	8-Feb-13	DZ#10	0	-
23-Mar-13	HC-1#9	0	-	16-Feb-13	BZ#1	0	-
23-Mar-13	HC-1#10	0.0035	+	16-Feb-13	BZ#2	0	-
23-Mar-13	HC-1#11	0	-	16-Feb-13	BZ#3	0	-
23-Mar-13	HC-1#12	0.0097	+	16-Feb-13	BZ#4	0	-
23-Mar-13	HC-1#13	0.0204	+	16-Feb-13	BZ#5	0	-
5-Jul-13	HC-3#1	0.0343	+	16-Feb-13	BZ#6	0	-
5-Jul-13	HC-3#2	0.0487	+	16-Feb-13	BZ#7	0	-
5-Jul-13	HC-3#3	0.0103	+	16-Feb-13	BZ#8	0	-

Gametocytemia (%) were obtained after counting a total 10,000 blood cells. “+” indicates detection of a PCR product of expected size; and “-” means no product of expected size was detected. Note: Sampling locations: ZZ, Zhangzhou, Fujian province; HC, Haicang, Xiamen, Fujian province; FZ, Fuzhou, Fujian province; GZ, Ganzhou, Jiangxi province; RJ, Ruijin, Jiangxi province; DZ, Dazhou, Sichuan Province; BZ, Binzhou of Shandong province. Note, some of the gametocytemia counts were reported previously [23].

### Monitoring seasonal infection dynamics in a Haicang chicken farm

We next used PCR and microscopy methods to monitor *Leucocytozoon* infection over a period of one year. We collected blood samples from chickens each month (missing February due to school winter break and national holidays), from October 2014 to September 2015. We then examined blood smears for gametocytes after counting at least 10,000 RBCs each and detected parasite DNA using the Ls-coxIIIF2/R2 PCR. We observed gametocytes in 59 out of 140 blood samples (42.1%) collected in 11 months (no positive smears were found in November, 2014), with infection rates ranging from 0% to 80% (**Table 2 and S1 Table**). When testing the samples using the Ls-coxIIIF2/R2 PCR primers, parasite DNAs were detected in 106 blood samples (75.7%) collected during all 12 months, with infection rates ranging from 33.3% to 100%. The data also showed that June had the highest infection rate by both methods. Three of the nine birds negative by blood smears in November 2014 were positive by the PCR method (**Table 2**).

**Table 2:** Monthly surveys of *Leucocytozoon* infections using microscopy of blood smear and the Ls-coxIIIF2/R2 PCR assay.

Month	No. birds exam	No. birds infected (by smear)	% positive by smear	No. birds infected (by PCR assay)	% positive by PCR assay
Oct., 2014	34	15	44.1	29	85.3
Nov., 2014	9	0	0	3	33.3
Dec., 2014	14	3	21.4	6	42.9
Jan., 2015	14	5	35.7	8	57.1
Mar., 2015	21	8	38.1	19	90.5
Apr., 2015	10	4	40	7	70
May., 2015	4	3	75	3	75
Jun., 2015	10	8	80	10	100
Jul., 2015	14	8	57.1	14	100
Aug., 2015	6	4	66.7	5	83.3
Sep., 2015	4	1	25	3	75
Sum	140	59	Ave 43.9	107	Ave 73.9

The animals were randomly selected from the farm at time of initial sampling. No sample was collected for February due to school winter break and national holidays.

### Monitoring infections in individual chickens over time

We also monitored infections of three infected chickens from a free-range farm in Haicang district, Xiamen city, in Fujian province over several months using the same microscopy of blood smears and the PCR method. These chickens were kept in a room at the university campus where no simuliid vectors were found and were sampled weekly in April and May and biweekly from June to October. All three chickens were gametocyte positive in April and became negative in late August by microscopy (**Table 3**). However, they were all positive in every sampling time by the Ls-coxIIIF2/R2 PCR method, suggesting that they could still transmit the parasite after several months of no active transmission.

**Table 3:** Weekly or biweekly infections of three chickens from a free-range farm in Xiamen, southern China, detected by blood smear or parasite-specific PCR.

	HC#1		HC#2		HC#3	
Date	Gametocytemia (%)	PCR detection	Gametocytemia (%)	PCR detection	Gametocytemia (%)	PCR detection
1-Apr-13	1	+	0	+	0.017	+
8-Apr-13	0.011	+	<0.001	+	0.041	+
16-Apr-13	0.02	+	0.007	+	0.163	+
22-Apr-13	0.012	+	0.008	+	0.101	+
29-Apr-13	0.004	+	0.027	+	0.066	+
6-May-13	<0.001	+	0	+	0.084	+
12-May-13	<0.001	+	0.002	+	0.045	+
17-May-13	0	+	<0.001	+	<0.001	+
21-May-13	<0.001	+	<0.001	+	0.009	+
28-May-13	<0.001	+	<0.001	+	0.057	+
7-Jun-13	0	+	<0.001	+	0.004	+
17-Jun-13	0	+	0	+	0.012	+
10-Jul-13	0	+	0	+	0.005	+
22-Jul-13	0	+	<0.001	+	0	+
5-Aug-13	0	+	0.001	+	0.001	+
15-Aug-13	0	+	0	+	0	+
3-Sep-13	0	+	0	+	0	+
18-Sep-13	0	+	0	+	0	+
2-Oct-13	0	+	0	+	0	+
14-Oct-13	0	+	0	+	0	+

Note: HC, Infected chickens from Haicang district, Xiamen city, Fujian province.”+”, indicates PCR products of expected size.

### Testing potential gametocidal drugs

**Fig 3:**
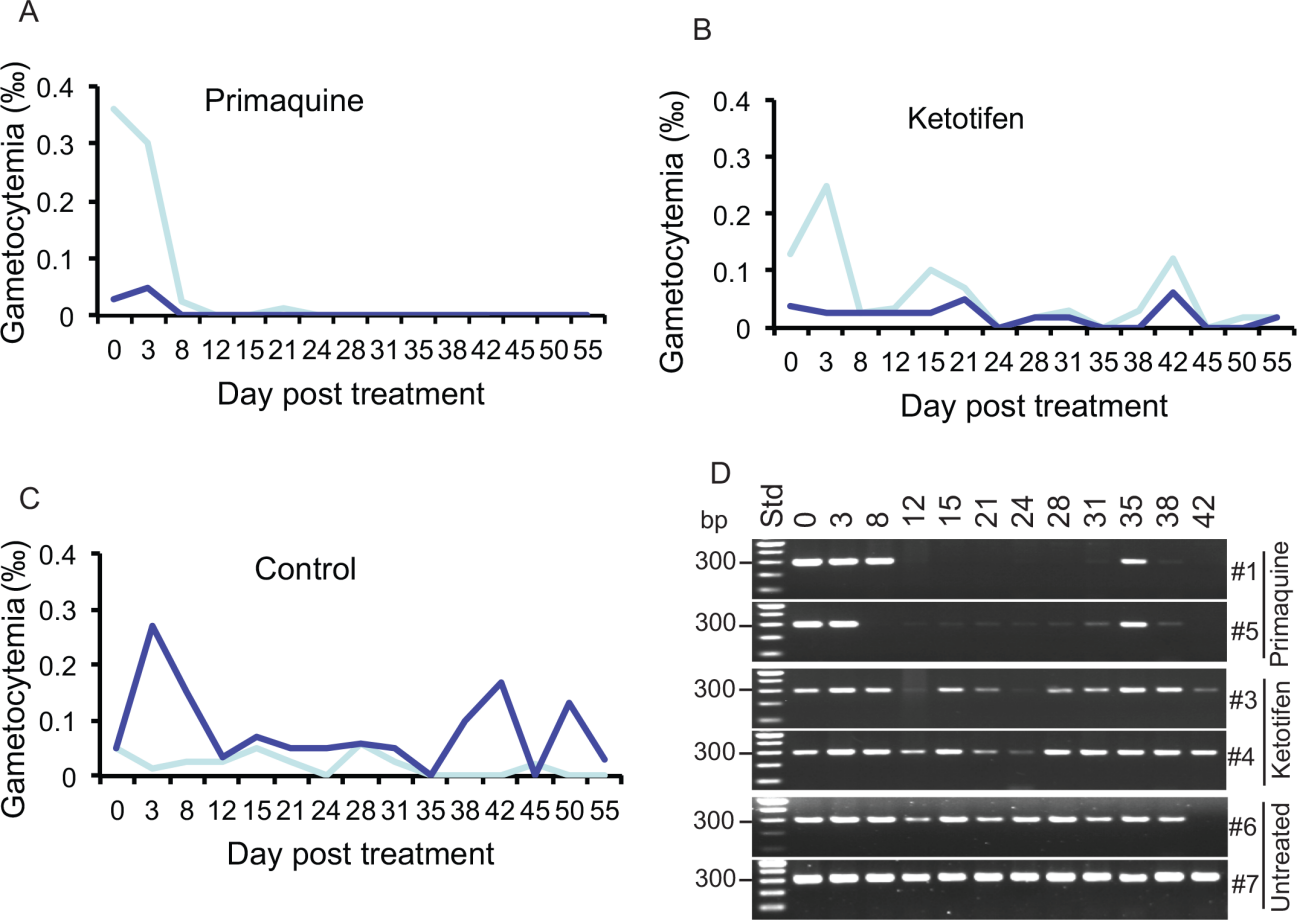
Detection of *Leucocytozoon* gametocytes or DNA after drug treatments. **A-C**, Curves of gametocytemia from two chickens after treatment (daily doses of 2 mg/kg for 14 days) with primaquine (**A**), ketotifen (**B**), or no-treatment controls (**C**). Each line in the graphs of A-C represents gametocytemia from an infected chicken. **D**, Agarose gels of PCR products from the Ls-coxIIIF2/R2 primers. #1 and #5 were treated with a dose of 2 mg/kg primaquine daily for 14 days; #3 and #4 were treated with a dose of 2 mg/kg ketotifen daily for 14 days; #6 and #7 were controls without treatment. All the chickens were from the Haicang (HC) farm. DNA sample preparation and PCR amplifications were as described in Materials and Methods.

In our previous study of *Plasmodium* parasites, we identified a class of tricyclic compounds that could block malaria transmission, some of which also had anti-gametocyte activities [37]. Here we were also interested in investigating whether these compounds had activities against gametocytes of *L*. *sabrazesi*. We tested primaquine (2 mg/kg), a compound that is known to kill gametocytes of malaria parasites, ketotifen (2 mg/kg) that had some activity against *Plasmodium* gametocytes, clomipramine hydrochloride (5 mg/kg), desipramine hydrochloride (5 mg/kg), sulfaquinoxaline (20 mg/kg), and pyrimethamine (3.5 mg/kg) (**Fig 3** and S2 **Table**). Primaquine clearly had activities against mature *L*. *sabrazesi* gametocytes. Mature gametocytes in chicken #5HC disappeared three days after initial primaquine administration and eight days in chicken #1HC, although a few gametocytes were found in #1HC 21 days after the treatment (**Fig 3A**). Gametocytes were present in the two chickens treated with ketotifen (#3HC and #4HC) and the two untreated controls (#6HC and #7HC) on most of the days during the periods of study (**Fig 3B** and **3C** **and S2 Table**). For these chickens, the gametocytemia changed (up and down) with time, suggesting clearing of old gametocytes (due to aging or immunity) and releasing new ones into the blood stream. Similarly, treatment with clomipramine hydrochloride, desipramine hydrochloride, sulfaquinoxaline, or pyrimethamine did not affect gametocytemia, suggesting no or limited effects of these drugs on *L*. *sabrazesi* gametocytes (**S2 Table**). We also used *coxIII* PCR to detect DNA in the drug-treated chickens (**Fig 3D and S2 Table**). The results from PCR detection also suggested that only primaquine had some effects on gametocytes, although the parasite positive dates did not match those of microscopic examination. For the primaquine-treated chickens, #1HC became negative on day 12, and #5HC became PCR negative on day 8 after administration of the drug (**Fig 3D**). However, they both became positive on day 35, suggesting that parasites were being released from internal tissues. Additionally, #5HC also had weak PCR bands from day 15–day 38. In summary, results from both blood smear examination and PCR DNA detection suggest that only primaquine has some effect on *L*. *sabrazesi* gametocytes, but may not have much effect on tissue stages. The suppression of gametocytemia by primaquine also suggests that this drug may be used to kill gametocyte and/or interrupt transmission of *L*. *sabrazesi*.

## Discussion

Accurate detection of *Leucocytozoon* infections is important for transmission control and disease management. Traditionally, diagnosis of *Leucocytozoon* infection is based on microscopic observation of mature gametocytes in blood smears. PCR amplification of parasite DNA, particularly when nested-PCR is performed [25, 27, 31], is expected to improve the sensitivity in detecting parasite infection. Indeed, PCR detections of blood protozoan infections, including *Leucocytozoon* infections, have been employed to survey prevalence in migrating birds and in insect vectors, to investigate parasite diversity, and to construct phylogenetic relationships [25, 27, 31]. In addition to amplification of parasite DNA from mature gametocytes, PCR can potentially detect DNA from any other stages of the parasite that are released into the blood stream, whereas microscopic examination is likely to miss immature gametocytes. Although various PCR methods have been reported to detect blood protozoan parasites in avian hosts, a method specifically to detect *L*. *sabrazesi* has not been described. To better monitor the transmission and infection of *L*. *sabrazesi* in domestic chickens in China, we tested two primer pairs derived from the parasite mitochondrial *cytb* and *coxIII* genes. Our results showed that both of the primer pairs were more sensitive in detecting *L*. *sabrazesi* infections than microscopic examination of blood smears, although we could not be sure that all the PCR positives (but smear negatives) were truly infected with the parasites. We decided to use the Ls-coxIII R2/R2 primers because they generally produced a single band of expected size, whereas the Ls-cytbF1/R1 primers sometimes had additional bands, including a band of ~350 bp in some samples (**Fig 2A**). The higher molecular weight band could be from some unknown organisms such as other blood parasites or from a *L*. *sabrazesi* strain that had a mutated allele, or it could simply be a non-specific product. To investigate whether the PCR products from microscopic negative samples were *L*. *sabrazesi* specific, we sequenced samples from 11 microscopic negative chickens and confirmed that all the sequences matched that of *L*. *sabrazesi*.

Testing samples using the Ls-coxIIIF2/R2 primers showed that 254 of the 255 microscopy positives (45 individual chickens in Table 1,59 samples from the monthly survey in S1 Table, and 150 samples in the drug treatment in S2 Table) were also positive by the *coxIII* PCR primers, suggesting a low false negative rate (false negative rate = 1-254/255 = 0.04%). From the same samples, 178 were microscopic gametocyte negative, and among the 178 samples, 88 (55.8%) were also PCR negative. These results suggested that PCR primers did not amplify the host gene because host DNA was present in each sample. The reason for the differences could be explained by the higher sensitivity of the PCR method, as indicated by testing a diluted DNA sample with known parasitemia (**Fig 1**). The PCR method could detect parasitemia as low as 2 parasites per 100,000 RBCs, while our counting standard was 10,000 RBCs, which was already more than the number of RBCs counted in most routine microscopic examinations. It is difficult to accurately estimate the false positive rate for the *coxIII* PCR assay because we do not know the real infection rate. We sequenced PCR products from 11 chickens that were microscopic negative, but were PCR positives. There were at least 16 individual parasite strains considering five mixed infections (Cloned sequences are not reliable because we cannot rule out errors introduced by PCR in the individual cloned sequences); all the PCR products were confirmed to be from *L*. *sabrazesi* (**S3 Fig**). These results as well as the mismatches in the primer sequences comparing with those of other representative species of blood parasites, particularly *L*. *caulleryi*, suggest that the *coxIII* PCR primers we used are relative specific for *L*. *sabrazesi*. To improve the sensitivity in detecting low-level infections, a nested-PCR method can be considered; however, nested-PCR is also very sensitive to laboratory contaminations because of two-round amplifications. The PCR method we established will be useful for monitoring the transmission of *L*. *sabranesi* in endemic regions in the future.

We investigated the infection rates each month for a full year and showed that chickens at a free-range farm in Haicang district, Xiamen city in Fujian province, were infected with *L*. *sabrazesi* year-round, with peak infection in June. The rainy season generally starts in late April and ends in August, and the average temperatures in the region range from 12°C in January/February to higher than 20°C from May to October. The higher infection rates in June–August were consistent with more rainfall and higher temperature that provide favorable conditions for vector reproduction and survival. The detection of year-round infection is also consistent with our previous observation of relapse of gametocytes; infected chickens could release gametocytes into circulation over a period of several months (**Table 3**) [23]. The previous observations of long-lasting gametocytes in the blood of infected chickens were further confirmed by our PCR survey monitoring DNA in the blood of three infected chickens. Our data suggest that transmission of the parasites to naïve chickens is likely to occur year-round on free-range farms in Xiamen or in southern China as long as insect vectors are available.

We also tested several compounds for treating *Leucocytozoon* infection, including two known antimalarial drugs (primaquine and pyrimethamine). Primaquine is an antimalarial drug that is known to kill gametocytes and liver stages of the human malaria parasites *P*. *falciparum* and *P*. *vivax*, and pyrimethamine is mostly used to treat blood asexual stages of malaria parasites [38, 39]. Other compounds we tested (ketotifen, desipramine hydrochloride, and clomipramine hydrochloride) were tricyclic antihistamines/antidepressants (TCAs); some of which were recently shown to have transmission blocking activities against malaria parasites, e.g., preventing oocyst development in a mosquito vector [37]. Our results here showed that primaquine had activity against mature *L*. *sabrazesi* gametocytes, but not pyrimethamine. Primaquine is a member of the 8-aminoquinoline drugs and is effective in treating liver and sexual stages of some *Apicomplexian* parasites, including species of *Plasmodium* [40] and *Leucocytozoon* parasites [11, 15], so results of activities against *L*. *sabrazesi* are consistent the previous observations. No obvious activities against *L*. *sabrazesi* gametocytes were observed for ketotifen, desipramine hydrochloride, clomipramine hydrochloride, or sulfaquinoxaline at the dosages tested. In malaria infection, the transmission blocking activities of these TCAs were based on counting oocysts in mosquito midguts; mature gametocytes may not be the target stages of the compounds. Indeed, ketotifen had weak activity against mature malaria gametocytes, but was very potent in preventing oocyst development in mosquitoes [37]. Additionally, the presence of the parasites in the blood may not reflect the viability/transmissibility of the gametocytes, and bloods with undetectable gametocytes may still allow transmission from an avian host to insect vectors. It would be interesting to test the activities of these compounds in inhibiting the stages of fertilization or early oocyst development in the insect vectors, although they do not have activities against *L*. *sabrazesi* mature gametocytes. A simple procedure to evaluate gametocytocidal and transmission blocking activities of artesunate in a *Plasmodium gallinaceum*-avian model was recently described [41]. Similar methods using *Simuliid* flies can be developed and used to test the activities of potential transmission blocking compounds against *Leucocytozoon* parasites; unfortunately, we currently do not have an insectary to raise the insect vectors.

This study develops a sensitive PCR method to detect *L*. *sabrazesi* infection in chicken. Using the PCR method and microscopic examination of blood smear, we also showed year-round infection of *L*. *sabrazesi* in Xiamen, southern China, although the infection rates are lower in the winter months. We also showed high prevalence of *L*. *sabrazesi* infection in four of the seven sampling locations in China. The data obtained in this study provide important information for formulating strategies for disease control and management.

## Supporting Information

S1 Fig Aligned DNA sequences amplified from microscopic negative blood samples using Ls-coxIIIF2/R2 primers. PCR products were sequenced directly without cloning into a plasmid vector.
(PDF)

S2 Fig Aligned DNA sequences of 24 individual clones originated from the 10 microscopic *Leucocytozoon sabrazesi* negative samples. The PCR products were first cloned into Pmd18-T vector, and DNAs from individual bacterial colonies were extracted and sequenced commercially. Name code: 0419#3p_C4, clone number 4 from sample 0419#3p.
(PDF)

S3 Fig Clustering of PCR amplified sequence fragments using Ls-coxIIIF2/R2 primers with those of representative blood parasites. The sequences are: *Leucocytozoon caulleryi* (Accession No. AB302215.1)*; Haemoproteus columbae* (NCBI accession No. FJ168562.1); *Plasmodium gallinaceum* (Accession No. AB250690.1), *Gallus gallus domesticus* (Accession No. KM096864.1), and the 11 amplified (uncloned) sequences. The sequences were aligned using Clustal W and clustered using the neighbor-joining method implemented in the program MEGA5 [42]. Bootstrap values higher than 60% are shown after 1,000 permutations.
(PDF)

S1 Table Monthly surveys of *Leucocytozoon* infections in a free-range farm from Haicang district, Xiamen city. (XLSX)

S2 Table Drug screening against gametocytes of *Leucocytozoon sabrazesi*. (XLSX)
