# Supplementary material for: Monitoring the Prevalence of Leucocytozoon sabrazesi in Southern China and Testing Tricyclic Compounds against Gametocytes
Source: PLoS One. 2016 Aug 29;11(8):e0161869. doi: 10.1371/journal.pone.0161869 (PMC5003344; doi:10.1371/journal.pone.0161869)
Supplement: S1 Fig — PCR products were sequenced directly without cloning into a plasmid vector. (PDF) [file pone.0161869.s001.pdf]

CLUSTAL O(1.2.2) multiple sequence alignment

```
0303#1          ACCCCAGAAGTAAGTTATGAATAATAATGCTTCTGATATAAT   TAAACTAAACATACCAGA
0303#2          ACCCCAGAAGTAAGTTATGAATAATAATGCTTCTGATATAAT   TAAACTAAACATACCAGA
0315#2          ACCCCAGAAGTAAGTTATGAATAATAATGCTTCTGATATAAT   TAAACTAAACATACCAGA
0408#3p         ACCCCAGAAGTAAGTTATGAATAATAATGCTTCTGATATAAT   TAAACTAAACATACCAGA
0608#4          ACCCCAGAAGTAAGTTATGAATAATAATGCTTCTGATATAAT   TAAACTAAACATACCAGA
0716#2          ACCCCAGAAGTAAGTTATGAATAATAATGCTTCTGATATAAT   TAAACTAAACATACCAGA
0716#5          ACCCCAGAAGTAAGTTATGAATAATAATGCTTCTGATATAAT(C)TAAACTAAACATACCAGA
0728#1          ACCCCAGAAGTAAGTTATGAATAATAATGCTTCTGATATAAT   TAAACTAAACATACCAGA
0728#5          ACCCCAGAAGTAAGTTATGAATAATAATGCTTCTGATATAAT   TAAACTAAACATACCAGA
0315#12         ACCCCAGAAGTAAGTTATGAATAATAATGCTTCTGATATAAT   TAAACTAAACATACCAGA
0419#3p         ACCCCAGAAGTAAGTTATGAATAATAATGCTTCTGATATAAT   TAAACTAAACATACCAGA
Ls-coxIII       ACCCCAGAAGTAAGTTATGAATAATAATGCTTCTGATATAAT   TAAACTAAACATACCAGA
                ******************************************   ******************

0303#1          AACTAATGAAGAAAATGTTGAGTATAAACATTCTCTAATTGAGTATAAGAATATTAATAA
0303#2          AACTAATGAAGAAAATGTTGAGTATAAACATTCTCTAATTGAGTATAAGAATATTAATAA
0315#2          AACTAATGAAGAAAATGTTGAGTATAAACATTCTCTAATTGAGTATAAGAATATTAATAA
0408#3p         AACTAATGAAGAAAATGTTGAGTATAAACATTCTCTAATTGAGTATAAGAATATTAATAA
0608#4          AACTAATGAAGAAAATGTTGAGTATAAACATTCTCTAATTGAGTATAAGAATATTAATAA
0716#2          AACTAATGAAGAAAATGTTGAGTATAAACATTCTCTAATTGAGTATAAGAATATTAATAA
0716#5          AACTAATGAAGAAAATGTTGAGTATAAACATTCTCTAATTGAGTATAAGAATATTAATAA
0728#1          AACTAATGAAGAAAATGTTGAGTATAAACATTCTCTAATTGAGTATAAGAATATTAATAA
0728#5          AACTAATGAAGAAAATGTTGAGTATAAACATTCTCTAATTGAGTATAAGAATATTAATAA
0315#12         AACTAATGAAGAAAATGTTGAGTATAAACATTCTCTAATTGAGTATAAGAATATTAATAA
0419#3p         AACTAATGAAGAAAATGTTGAGTATAAACATTCTCTAATTGAGTATAAGAATATTAATAA
Ls-coxIII       AACTAATGAAGAAAATGTTGAGTATAAACATTCTCTAATTGAGTATAAGAATATTAATAA
                ************************************************************

0303#1          TGTAATTAG   ATTTAATGAGAATAATATTCCAACTGAAAAGTATTTTAAAGATGA   AGCATA
0303#2          TGTAATTAG   ATTTAATGAGAATAATATTCCAACTGAAAAGTATTTTAAAGATGA   AGCATA
0315#2          TGTAATTAG   ATTTAATGAGAATAATATTCCAACTGAAAAGTATTTTAAAGATGA   AGCATA
0408#3p         TGTAATTAA   ATTTAATGAGAATAATATTCCAACTGAAAAGTATTTTAAAGATGT   AGCATA
0608#4          TGTAATTAA   ATTTAATGAGAATAATATTCCAACTGAAAAGTATTTTAAAGATGT   AGCATA
0716#2          TGTAATTAA(G)ATTTAATGAGAATAATATTCCAACTGAAAAGTATTTTAAAGATGT(A)AGCATA
0716#5          TGTAATTAA   ATTTAATGAGAATAATATTCCAACTGAAAAGTATTTTAAAGATGT   AGCATA
0728#1          TGTAATTAA   ATTTAATGAGAATAATATTCCAACTGAAAAGTATTTTAAAGATGT(A)AGCATA
0728#5          TGTAATTAA   ATTTAATGAGAATAATATTCCAACTGAAAAGTATTTTAAAGATGT   AGCATA
0315#12         TGTAATTAA   ATTTAATGAGAATAATATTCCAACTGAAAAGTATTTTAAAGATGA(T)AGCATA
0419#3p         TGTAATTAA   ATTTAATGAGAATAATATTCCAACTGAAAAGTATTTTAAAGATGA(T)AGCATA
Ls-coxIII       TGTAATTAA   ATTTAATGAGAATAATATTCCAACTGAAAAGTATTTTAAAGATGT   AGCATA
                ********    ********************************************    ******
```

0303#1	TAATGATGTAATACTTGGATAA
0303#2	TAATGATGTAATACTTGGATAA
0315#2	TAATGATGTAATACTTGGATAA
0408#3p	TAATGATGTAATACTTGGATAA
0608#4	TAATGATGTAATACTTGGATAA
0716#2	TAATGATGTAATACTTGGATAA
0716#5	TAATGATGTAATACTTGGATAA
0728#1	TAATGATGTAATACTTGGATAA
0728#5	TAATGATGTAATACTTGGATAA
0315#12	TAATGATGTAATACTTGGATAA
0419#3p	TAATGATGTAATACTTGGATAA
Ls-coxIII	TAATGATGTAATACTTGGATAA
	**********************
