# Supplementary material for: Monitoring the Prevalence of Leucocytozoon sabrazesi in Southern China and Testing Tricyclic Compounds against Gametocytes
Source: PLoS One. 2016 Aug 29;11(8):e0161869. doi: 10.1371/journal.pone.0161869 (PMC5003344; doi:10.1371/journal.pone.0161869)
Supplement: S2 Fig — The PCR products were first cloned into Pmd18-T vector, and DNAs from individual bacterial colonies were extracted and sequenced commercially. Name code: 0419#3p_C4, clone number 4 from sample 0419#3p. (PDF) [file pone.0161869.s002.pdf]

CLUSTAL O(1.2.2) multiple sequence alignment

```
0419#3p_C4      AGCTATACCTTCATTACTTAATGGATAAGGTGATAGAATGAAATGGAATATACCCCAGAA
0419#3p_C1      AACTATACCTTCATTACTTAATGGATAGGGTGATAGAATGAAATGGAATATACCCCAGAA
0303#1_C2       AACTATACCTTCATTACTTAATGGATAAGGTGATAGAATGAAATGGAATATACCCCAGAA
0315#2_C2       AACTATACCTTCATTACTTAATGGATAAGGTGATAGAATGAAATGGAATATACCCCAGAA
0315#2_C3       AACTATACCTTCATTACTTAATGGATAAGGTGATAGAATGAAATGGAATATACCCCAGAA
0716#2_C3       AACTATACCTTCATTACTTAATGGATAAGGTGATAGAATGAAATGGAATATACCCCAGAA
0303#1_C3       AACTATACCTTCATTACTTAATGGATAAGGTGATAGAATGAAATGGAATATACCCCAGAA
0716#2_C4       AACTATACCTTCATTACTTAATGGATAAGGTGATAGAATGAAATGGAATATACCCCAGAA
0315#12_C5      AACTATACCTTCATTGCTTAATGGATAAGGTGATAGAATAAAATGGAATATACCCCAGAA
0728#1_C9       AACTATACCTTCATTGCTTAATGGATAAGGTGATAGAATAAAATGGAATATACCCCAGAA
0728#1_C10      AACTATACCTTCATTGCTTAATGGATAAGGTGATAGAATAAAATGGAATATACCCCAGAA
0716#2_C5       TACTATACCTTCATTACTTAATGGATAAGGTGATAGAATAAAATGGAATATACCCCAGAA
0408#3p_C3      TACTATACCTTCATTACTTAATGGATAAGGTGATAGAATAAAATGGAATATACCCCAGAA
0728#1_C8       TACTATACCTTCATTACTTAATGGATAAGGTGATAGAATAAAATGGAATATACCCCAGAA
0419#3p_C8      AACTATACCTTCATTACTTAATGGATAAGGTGATAGAATAAAATGGAATATACCCCAGAA
0728#1_C2       TACTATACCTTCGTTACTTAATGGATAAGGTGATAGAATAAAATGGAATATACCCCAGAA
0608#4_C1       TACTATACCCTCATTACTTAATGGATAAGGTGATAGAATAAAATGGAATATACCCCAGAA
0419#3p_C2      TACTATACCTTCATTACTTAATGGATAAGGTGATAGAATAAAATGGAATATACCCCAGAA
0608#4_C2       TACTATACCTTCATTACTTAATGGATAAGGTGATAGAATAAAATGGAATATACCCCAGAA
0716#5_C1       TACTATACCTTCATTACTTAATGGATAAGGTGATAGAATAAAATGGAATATACCCCAGAA
0728#1_C1       TACTATACCTTCATTACTTAATGGATAAGGTGATAGAATAAAATGGAATATACCCCAGAA
0728#5_C3       TACTATACCTTCATTACTTAATGGATAAGGTGATAGAATAAAATGGAATATACCCCAGAA
0419#3p_C7      TACTATACCTTCATTACTTAATGGATAAGGTGATAGAATAAAATGGAATATACCCCAGAA
0315#12_C1      TACTATACCTTCATTACTTAATGGATAAGGTGATAGAATGAAATGGAATATACCCCAGAA
Ls-coxIII       TACTATACCTTCATTACTTAATGGATAAGGTGATAGAATAAAATGGAATATACCCCAGAA
                  ******* ** ** *********** *********** ********************

0419#3p_C4      GTAAGTTATGAATAATAATGCTTCTGATATAATTAAACTAAACATACCAGAAACTAATGA
0419#3p_C1      GTAAGTTATGAATAATAATGCTTCTGATATAATTAAACTAAACATACCAGAAACTAATGA
0303#1_C2       GTAAGTTATGAATAATAATGCTTCTGATATAATTAAACTAAACATACCAGAAACTAATGA
0315#2_C2       GTAAGTTATGAATAATAATGCTTCTGATATAATTAAACTAAACATACCAGAAACTAATGA
0315#2_C3       GTAAGTTATGAATAATAATGCTTCTGATATAATTAAACTAAACATACCAGAAACTAATGA
0716#2_C3       GTAAGTTATGAATAATAATGCTTCTGATATAATTAAACTAAACATACCAGAAACTAATGA
0303#1_C3       GTAAGTTATGAATAATAATGCTTCTGATATAATTAAACTAAACATACCAGAAACTAATGA
0716#2_C4       GTAAGTTATGAATAATAATGCTTCTGATATAATTAAACTAAACATACCAGAAACTAATGA
0315#12_C5      GTAAGTTATGAATAATAATGCTTCTGATATAATTAAACTAAACATACCAGAAACTAATGA
0728#1_C9       GTAAGTTATGAATAATAATGCTTCTGATATAATTAAACTAAACATACCAGAAACTAATGA
0728#1_C10      GTAAGTTATGAATAATAATGCTTCTGATATAATTAAACTAAACATACCAGAAACTAATGA
0716#2_C5       GTAAGTTATGAATAATAATGCTTCTGATATAATTAAACTAAACATACCAGAAACTAATGA
0408#3p_C3      GTAAGTTATGAATAATAATGCTTCTGATGTAATTAAACTAAACATACCAGAAACTAATGA
0728#1_C8       GTAAGTTATGAATAATAGTGCTTCTGATATAATTAAACTAAACATACCAGAAACTAATGA
```

```
0419#3p_C8      GTAAGTTATGAATAATAATGCTTCTGATATAATTAAACTAAACATACCAGAAACTAATGA
0728#1_C2       GTAAGTTATGAATAATAATGCTTCTGATATAATTAAACTAAACATACCAGAAACTAATGA
0608#4_C1       GTAAGTTATGAATAATAATGCTTCTGATATAATTAAACTAAACATACCAGAAACTAATGA
0419#3p_C2      GTAAGTTATGAATAATAATGCTTCTGATATAATTAAACTAAACATACCAGAAACTAATGA
0608#4_C2       GTAAGTTATGAATAATAATGCTTCTGATATAATTAAACTAAACATACCAGAAACTAATGA
0716#5_C1       GTAAGTTATGAATAATAATGCTTCTGATATAATTAAACTAAACATACCAGAAACTAATGA
0728#1_C1       GTAAGTTATGAATAATAATGCTTCTGATATAATTAAACTAAACATACCAGAAACTAATGA
0728#5_C3       GTAAGTTATGAATAATAATGCTTCTGATATAATTAAACTAAACATACCAGAAACTAATGA
0419#3p_C7      GTAAGTTATGAATAATAATGCTTCTGATATAATTAAACTAAACATACCAGAAACTAATGA
0315#12_C1      GTAAGTTATGAATAATAATGCTTCTGATATAATTAAACTAAACATACCAGAAACTAATGA
Ls-coxIII       GTAAGTTATGAATAATAATGCTTCTGATATAATTAAACTAAACATACCAGAAACTAATGA
                **************** ********** ********************************

0419#3p_C4      AGAAAATGTTGAGTATAAACATTCTCTAATTGAGTATAAGAATATTAATAATGTAATTAG
0419#3p_C1      AGAAAATGTTGAGTATAAACATTCTCTAATTGAGTATAAGAATATTAATAATGTAATTAG
0303#1_C2       AGAAAATGTTGAGTATAAACATTCTCTAATTGAGTATAAGAATATTAATAATGTAATTAG
0315#2_C2       AGAAAATGTTGAGTATAAACATTCTCTAATTGAGTATAAGAATATTAATAATGTAATTAG
0315#2_C3       AGAAAATGTTGAGTATAAACATTCTCTAATTGAGTATAAGAATATTAATAATGTAATTAG
0716#2_C3       AGAAAATGTTGAGTATAAACATTCTCTAATTGAGTATAAGAATATTAATAATGTAATTAG
0303#1_C3       AGAAAATGTTGAGTATAAACATTCTCTAATTGAGTATAAGAATATTAATAATGTAATTAG
0716#2_C4       AGAAAATGTTGAGTATAAACATTCTCTAATTGAGTATAAGAATATTAATAATGTAATTAG
0315#12_C5      AGAAAATGTTGAGTATAAACATTCTCTAATTGAGTATAAGAATATTAATAATGTAATTAA
0728#1_C9       AGAAAATGTTGAGTATAAACATTCTCTAATTGAGTATAAGAATATTAATAATGTAATTAA
0728#1_C10      AGAAAATGTTGAGTATAAACATTCTCTAATTGAGTATAAGAATATTAATAATGTAATTAA
0716#2_C5       AGAAAATGTTGAGTATAAACATACTCTAATTGAGTATAAGAATATTAATAATGTAATTAG
0408#3p_C3      AGAAAATGTTGAGTATAAACATTCTCTAATTGAGTATAAGAATATTAATAATGTAATTAA
0728#1_C8       AGAAAATGTTGAGTATAAACATTCTCTAATTGAGTATAAGAATATTAATAATGTAATTAA
0419#3p_C8      AGAAAATGTTGAGTATAAACATTCTCTAATTGAGTATAAGAATATTAATAATGTAATTAA
0728#1_C2       AGAAAATGTTGAGTATAAACATTCTCTAATTGAGTATAAGAATATTAATAATGTAATTAA
0608#4_C1       AGAAAATGTTGAGTATAAACATTCTCTAATTGAGTATAAGAATATTAATAATGTAATTAA
0419#3p_C2      AGAAAATGTTGAGTACAAACATTCTCTAATTGAGTATAAGAATATTAATAATGTAATTAA
0608#4_C2       AGAAAATGTTGAGTATAAACATTCTCTAATTGAGTATAAGAATATTAATAATGTAATTAA
0716#5_C1       AGAAAATGTTGAGTATAAACATTCTCTAATTGAGTATAAGAATATTAATAATGTAATTAA
0728#1_C1       AGAAAATGTTGAGTATAAACATTCTCTAATTGAGTATAAGAATATTAATAATGTAATTAA
0728#5_C3       AGAAAATGTTGAGTATAAACATTCTCTAATTGAGTATAAGAATATTAATAATGTAATTAA
0419#3p_C7      AGAAAATGTTGAGTATAAACATTCTCTAATTGAGTATAAGAATATTAATAATGTAATTAA
0315#12_C1      AGAAAATGTTGAGTATAAACATTCTCTAATTGAGTATAAGAATATTAATAATGTAATTAA
Ls-coxIII       AGAAAATGTTGAGTATAAACATTCTCTAATTGAGTATAAGAATATTAATAATGTAATTAA
                *************** ****** ************************************

0419#3p_C4      ATTTAATGAGAATAATATTCCAACTGAAAAGTATTTTAAAGATGAAGCATATAATGATGT
0419#3p_C1      ATTTAATGAAAATAATATTCCAACTGAAAAGTATTTTAAAGATGAAGCATATAATGATGT
0303#1_C2       ATTTAATGAGAATAATATTCCAACTGAAAAGTATTTTAAAGATGAAGCATATAATGATGT
0315#2_C2       ATTTAATGAGAATAATATTCCAACTGAAAAGTATTTTAAAGATGAAGCATATAATGATGT
```

0315#2_C3	ATTTAATGAGAATAATATTCCAACTGAAAAGTATTTTAAAGATGAAGCATATAATGATGT
0716#2_C3	ATTTAATGAGAATAATATTCCAACTGAAAAGTATTTTAAAGATGAAGCATATAATGATGT
0303#1_C3	ATTTAATGAGAATAATATTCCAACTGAAAAGTATTTTAAAGATGAAGCATATAATGATGT
0716#2_C4	ATTTAATGAGAATAATATTCCAACTGAAAAGTATTTTAAAGATGAAGCATATAATGATGT
0315#12_C5	GTTTAATGAGAATAATATTCCAACTGAAAAGTATTTTAAAGATGAAGCATATAATGATGT
0728#1_C9	GTTTAATGAGAATAATATTCCAACTGAAAAGTATTTTAAAGATGAAGCATATAATGATGT
0728#1_C10	GTTTAATGAGAATAATATTCCAACTGAAAAGTACTTTAAAGATGAAGCATATAATGATGT
0716#2_C5	ATTTAATGAGAATAATATTCCAACTGAAAAGTATTTTAAAGATGAAGCATATAATGATGT
0408#3p_C3	GTTTAATGAGAATAATATTCCAACTGAAAAGTATTTTAAAGATGAAGCATATAATGATGT
0728#1_C8	ATTTAATGAGAATAATATTCCAACTGAAAAGTATTTTAAAGATGTAGCATATAATGATGT
0419#3p_C8	ATTTAATGAGAATAATATTCCAACTGAAAAGTATTTTAAAGATGTAGCATATAATGATGT
0728#1_C2	ATTTAATGAGAATAATATTCCAACTGAAAAGTATTTTAAAGATGTAGCATATAATGATGT
0608#4_C1	ATTTAATGAGAATAATATTCCAACTGAAAAGTATTTTAAAGATGTAGCATATAATGATGT
0419#3p_C2	ATTTAATGAGAATAATATTCCAACTGAAAAGTATTTTAAAGATGTAGCATATAATGATGT
0608#4_C2	ATTTAATGAGAATAATATTCCAACTGAAAAGTATTTTAAAGATGTAGCATATAATGATGT
0716#5_C1	ATTTAATGAGAATAATATTCCAACTGAAAAGTATTTTAAAGATGTAGCATATAATGATGT
0728#1_C1	ATTTAATGAGAATAATATTCCAACTGAAAAGTATTTTAAAGATGTAGCATATAATGATGT
0728#5_C3	ATTTAATGAGAATAATATTCCAACTGAAAAGTATTTTAAAGATGTAGCATATAATGATGT
0419#3p_C7	ATTTAATGAGAATAATATTCCAACTGAAAAGTATTTTAAAGATGTAGCATATAATGATGT
0315#12_C1	ATTTAATGAGAATAATATTCCAACTGAAAAGTATTTTAAAGATGTAGCATATAATGATGT
Ls-coxIII	ATTTAATGAGAATAATATTCCAACTGAAAAGTATTTTAAAGATGTAGCATATAATGATGT
	******** ********************** ********** ***************

0419#3p_C4	AATACTTGGATAA
0419#3p_C1	AATACTTGGATAA
0303#1_C2	AATACTTGGATAA
0315#2_C2	AATACTTGGATAA
0315#2_C3	AATACTTGGATAA
0716#2_C3	AATACTTGGATAA
0303#1_C3	AATACTTGGATAG
0716#2_C4	AATACTTGGATAG
0315#12_C5	AATACTTGGATAA
0728#1_C9	AATACTTGGATAA
0728#1_C10	AATACTTGGATAA
0716#2_C5	AATACTTGGATAA
0408#3p_C3	AATACTTGGATAA
0728#1_C8	AATACTTGGATAA
0419#3p_C8	AATACTTGGATAA
0728#1_C2	AATACTTGGATAA
0608#4_C1	AATACTTGGATAA
0419#3p_C2	AATACTTGGATAA
0608#4_C2	AATACTTGGATAA
0716#5_C1	AATACTTGGATAA
0728#1_C1	AATACTTGGATAA

0728#5_C3	AATACTTGGATAA
0419#3p_C7	AATACTTGGATAA
0315#12_C1	AATACTTGGATAA
Ls-coxIII	AATACTTGGATAA
	************
