# Supplementary figures and images for: Monitoring the Prevalence of Leucocytozoon sabrazesi in Southern China and Testing Tricyclic Compounds against Gametocytes
Source: PLoS One. 2016 Aug 29;11(8):e0161869. doi: 10.1371/journal.pone.0161869 (PMC5003344; doi:10.1371/journal.pone.0161869)

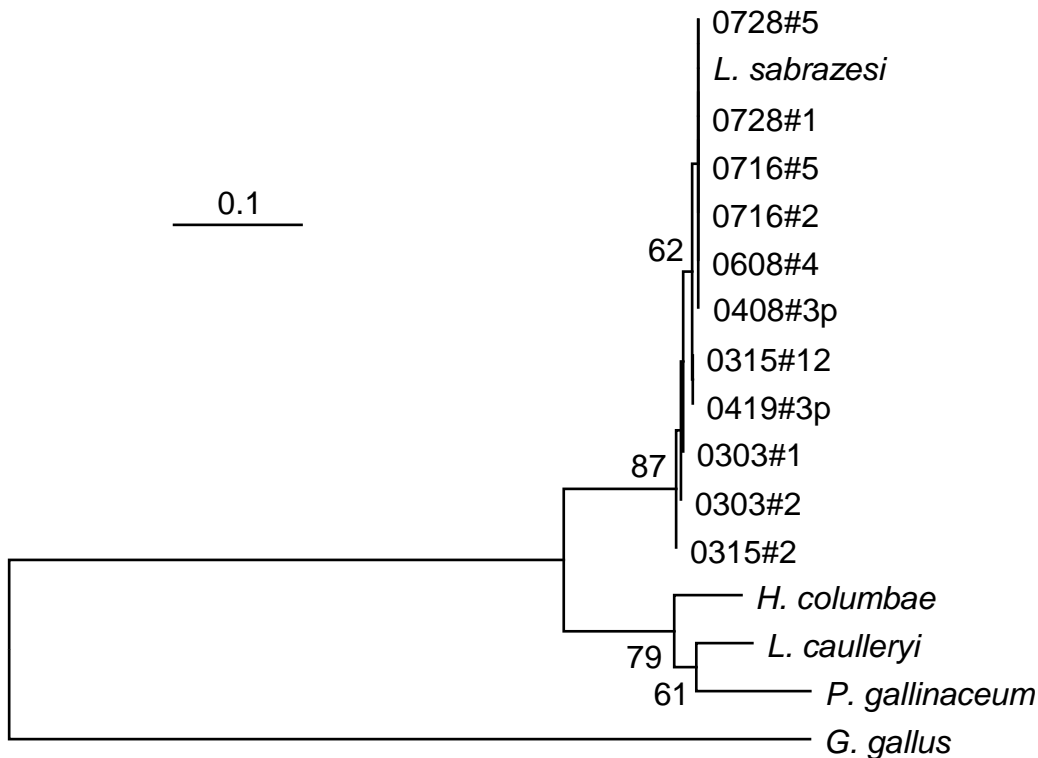

Supplement: S3 Fig — The sequences are: Leucocytozoon caulleryi (Accession No. AB302215.1); Haemoproteus columbae (NCBI accession No. FJ168562.1); Plasmodium gallinaceum (Accession No. AB250690.1), Gallus gallus domesticus (Accession No. KM096864.1), and the 11 amplified (uncloned) sequences. The sequences were aligned using Clustal W and clustered using the neighbor-joining method implemented in the program MEGA5 [42]. Bootstrap values higher than 60% are shown after 1,000 permutations. (PDF) [file pone.0161869.s003.pdf]
